# Voices of the accelerated: key themes when considering implementation of an accelerated medical school program

**DOI:** 10.1080/10872981.2024.2385666

**Published:** 2024-08-04

**Authors:** Francesco Satriale, Arianna Winchester, Michael Partin

**Affiliations:** a Department of Family and Community Medicine, Penn State Health Milton S Hershey Medical Center, Hershey, PA, USA; b Department of Otolaryngology-Head and Neck Surgery, New York University Grossman School of Medicine, New York, NY, USA

**Keywords:** Accelerated medical school, debt reduction, burnout reduction, physician shortage, residency preparedness, medical student mentorship, self-directed learning, professional identity, well-being

## Abstract

In this rapid communication, accelerated undergraduate medical education is examined using prior literature as well as experiences of those who have completed or are in the process of completing accelerated medical curricula. The Consortium of Accelerated Medical Pathway Programs (CAMPP) hosts an annual multi-institutional conference for all its members. During the meeting in July 2023, a virtual panel was convened from multiple constituent programs (*N* = 4) including medical students (*N* = 2), resident physicians (*N* = 4), and faculty (*N* = 2). Panel participants represented current learners or graduates from accelerated pathways of varying specialties (*N* = 5) to share firsthand experiences about acceleration to an audience representing over 25 medical schools. Five key themes were identified for accelerated students and trainees: Reduced debt as motivating factor to accelerate, Feeling prepared for residency, Ideal accelerated students are driven, Ability to form early professional relationships, and Less time for additional clinical experiences. Discourse from the CAMPP panel can inform current and developing accelerated programs at institutions looking to create or improve accelerated learning.

## Introduction

Three-year accelerated programs in undergraduate medical education are quickly growing in popularity. While the model has been used in other countries for decades (e.g., Canada and the United Kingdom), the support for accelerating undergraduate medical education (UME) has greatly increased in the past several years [1]. In 2015, the Consortium of Accelerated Medical Pathway Programs (CAMPP) was established with 8 member schools. In 2023, there are 31 member schools representing greater than a 3-fold increase [2]. The development of accelerated options is thought to be in response to increasing physician shortages, rising student debt, and the increasing burden of physician burnout. Data from 2021 estimates a shortage of between 37,800 and 124,000 physicians by 2034, with shortfalls in both primary and specialty care [3]. It is clear there is a need to increase the efficiency with which we train new physicians to meet the healthcare needs of the United States (US), and there is building evidence that accelerated UME is equally as effective as the traditional timeline. Accelerated programs abroad have shown no decrease in physician quality at any level of training [4], and initial studies in the US have been consistent. At New York University Grossman School of Medicine (NYUGSOM), one of the founding schools of CAMPP, no difference in trainees from the 3 or 4 year program has been identified based on clinical skills or overall intern ratings [5]. Additionally, accelerated students have voiced similar levels of satisfaction with their education, preparedness for residency, and graduate with less student debt [6]. As more domestic accelerated programs are developed, more studies are required to solidify these benefits.

## CAMPP panel discussion and qualitative analysis of themes

To better understand dynamics across multiple accelerated programs, CAMPP hosts an annual multi-institutional conference for all its members. During the meeting in July 2023, a virtual panel was convened from multiple constituent programs (*N* = 4) including medical students (*N* = 2), resident physicians (*N* = 4), and faculty (*N* = 2). Panel participants represented current learners or graduates from accelerated pathways of varying specialties (*N* = 5) to share firsthand experiences about acceleration to an audience representing over 25 medical schools. This rapid communication aims to examine learner and graduate perspectives shared during the panel to enhance current understanding and promote best practices for accelerated medical school pathways.

Themes regarding the motivation to accelerate and common shared experiences prior to medical school arose during the discussion (Table 1). Several panelists shared their experience entering medicine as a second or third career and being a ‘non-traditional’ applicant. Multiple panelists reported early interest in their desired specialty, oftentimes before medical school matriculation, which motivated them to pursue a shortened UME. Students and graduates also identified savings on the cost of tuition to be of considerable value. ***One panelist noted that ‘…the fact that I was able to cut that debt down is significant for me … it just made it all the more pleasant of an option.’***

**Table 1.** Key themes related to accelerated medical education identified during CAMPP panel discussion.

	Representative Quotes
Theme 1: Debt Reduction	*“I’ve spent a couple decades doing other things before medical school and so having to stop my life and stop making an income, you know is a big deal so the fact that I was able to cut that debt down is significant for me. I’m not going to say it was a bar that I wouldn’t have gone had this opportunity not presented itself but to me it just made it all the more pleasant of an option to be able to go ahead and make this change in my life.”*
Theme 2: Feeling Prepared for Residency	*“I can remember back to the past year when any of my colleagues who are doing traditional four-year medical school had a lot of time off. Being a part of a 3-year program you’re able to [forgo the fourth year] and once you matriculate into residency being able to see you can work alongside those who did four years and be essentially indistinguishable in terms of the work that you’re doing and how well you’re doing is rewarding.”*
Theme 3: Ideal Accelerated Student Qualities	*“ … qualities that I look for in SubIs or my colleagues [a good accelerated student] is someone who is hard working, who can work on a team because residency is very team-based. Someone obviously dedicated to studying and to knowing things, but more than anything wants to be there and wants to work.”*
Theme 4: Developing Early Relationships	*“I would say that with my experience it has kind of come full circle now that I am doing residency interviews and interacting with students that are starting the accelerated program. I agree there’s a risk that that’s involved but when you know these students for 3 years and you know their work ethic, you know their potential. I almost feel like there’s a sense of security from a GME standpoint knowing who’s coming into your residency program.”*
Theme 5: Less Time for Additional Experiences	*“People in my field tend to do a handful of away rotations, so usually you do at least your home AI, which I got to do, and then you do another local SubI and then at least one or two further away or other SubIs. So two to three kind of additional months spent on an ENT service learning about the field that my four year colleagues got to do and I didn’t, so I did feel like there was some more exposure [for four year students] but that’s just specifically because we don’t have a lot of ENT exposure, but that is definitely a difference that I felt.”*

Residency programs and/or health systems may raise concern about the preparedness of accelerated students to perform at an equivalent clinical level of traditionally trained students. However, both trainees and their mentors refuted this worry. ***The overwhelming majority of resident or graduate panelists felt well prepared for residency*** with one panelist stating, ‘…being able to see you can work alongside those who did four years and be essentially indistinguishable in terms of the work that you’re doing and how well you’re doing is rewarding.’ A former accelerated program graduate who is now in the role of associate program director shared their opinion of accepting other accelerated trainees into residency, stating, ‘when you know these students for 3 years, you know their work ethic, and you know their potential, I almost feel there’s a sense of security from a GME standpoint.’ In other words, accelerated training allows faculty to spend the entire length of UME developing a mentoring relationships and scholarly collaborations before residency even begins.

Participants shared that the ideal accelerated student possesses traits of self-directed learning, resilience, flexibility, efficiency, and being proactive. ***One panelist noted, ‘[an accelerated student] is someone who is hard working, who can work on a team because residency is very team-based.’*** Since accelerated students often make up a subsection of a medical school’s overall class, panelists agreed about the importance of programs assisting in development of a ‘sub-community’ of support within the accelerated cohort. Successful accelerated programs also reported the importance of identifying faculty champions outside of each student’s specialty who are focused on supporting their nontraditional journey. Concerns were shared about the potential for accelerated students to discount experiences outside of their specialty, however panelists highlighted the important guidance to ‘not lose sight that every clerkship and experience can give you something’ regardless of what field it is learned from, and ‘to have an eye to the future.’ ***As a result, students can develop their professional identity both within and outside of their specialty from day one of medical school.***

Panelists were asked about the potential drawbacks of accelerating and noted the difficulty of pursuing away rotations, especially in traditionally more competitive specialties like Otolaryngology and Ophthalmology. Further, students are often tasked with completing tasks such as residency applications, board exams, and rotations out of the traditional order. Panelists noted that early mentorship and guidance from specialty-specific faculty can help mitigate these drawbacks.

## Lessons learned

Overall, the discourse of the CAMPP panel highlighted that accelerated students are often ‘non-traditional’ applicants, motivated by reduction in debt and cost savings, and felt clinically indistinguishable from fellow traditional students at the start of residency. ***Best practices for programs include fostering a sense of wellbeing and community amongst the cohort*** and providing intentional specialty specific mentorship from day one of medical school. The primary strength of this panel review is the delineation of firsthand perspectives of accelerated students and graduates, which are sparse in the literature. Drawbacks include the potential for reporting bias amongst the panelists, and the fact that there were a small number of panelists participating. Reflections from this panel can inform current accelerated programs and assist in the development of future accelerated pathways at other organizations.

## Supplementary Material

Supplemental Material
