# Supplementary material for: Voices of the accelerated: key themes when considering implementation of an accelerated medical school program
Source: Med Educ Online. 2024 Aug 4;29(1):2385666. doi: 10.1080/10872981.2024.2385666 (PMC11299450; doi:10.1080/10872981.2024.2385666)
Supplement: Supplemental Material [file ZMEO_A_2385666_SM3602.docx]

**Supplemental Material**

**Supp. Table 1.** Early Faculty and Trainee Panel Participants’ Level of Training and Specialties.

| Participant Level of Training | Specialty |
| --- | --- |
| Faculty | Family Medicine |
| Resident (PGY-2) | Family Medicine |
| Resident (PGY-3) | Otolaryngology |
| Resident (PGY-3) | Ophthalmology |
| Resident (PGY-3) | Dermatology |
| Medical Student (MS-1) | Psychiatry |
| Medical Student (MS-3) | Family Medicine |
| Faculty | Dermatology |

*All virtual panelists* Panel size N=8, residents N = 4, faculty N = 2, medical students N = 2, institutions represented N = 4, specialties N = 5, primary care N = 3. Panelists represented 4 CAMPP constituent programs.
